# Pan-Genome Analysis of the *Tubulin* Gene Family Reveals Candidates for Fiber Strength in *Gossypium barbadense*

**DOI:** 10.3390/genes17080873

**Published:** 2026-07-27

**Authors:** Yajie Duan, Ruihong Zeng, Yongsheng Cai, Xiaoju Liu, Fenglei Sun

**Affiliations:** 1 College of Ecological Landscape Engineering, Xinjiang Agricultural Vocational and Technical University, Changji 831100, China; dyj547525782@126.com (Y.D.); z741119789@163.com (R.Z.); liuxiaoju317@163.com (X.L.); 2 College of Agronomy, Xinjiang Agricultural University, Urumqi 830052, China; cys0620@126.com; 3 National Cotton Engineering Technology Research Center, Cotton Research Institute of Xinjiang Uyghur Autonomous Region Academy of Agricultural Sciences, Urumqi 830091, China

**Keywords:** Pan-genome, *Tub*, fiber strength, *Gossypium barbadense*, cotton, cytoskeleton

## Abstract

**Background/Objectives**: *Tubulins* (*Tub*) are central components of microtubules, but intraspecific variation and developmental expression of the *Tub* family in *Gossypium barbadense* remain poorly characterized. This study aimed to characterize the *GbTub* family using a pan-genome framework and identify candidates associated with fiber development and strength. **Methods**: A total of 50 *GbTub* genes were identified in the *G. barbadense* 3-79 reference genome, and their orthologous presence–absence patterns were subsequently assessed across 12 additional *G. barbadense* accessions. Phylogenetic, presence–absence variation (PAV), Ka/Ks, structural variation (SV), RNA-seq, RT-qPCR, co-expression, and GO enrichment analyses were integrated. **Results**: Among the 50 reference-defined *GbTub* genes, 43 were classified as core genes, 6 as near-core genes, and 1 as an accessory gene, and the encoded proteins were classified into α-, β-, and *γ-tubulin* clades. All genes showed Ka/Ks < 1. Twenty-three *GbTub* genes differed between the fiber-strength-contrasting accessions 5917 and PimaS-7, and representative expression trends were supported by RT-qPCR. Network analysis prioritized 10 *GbTub* candidates based on degree centrality. *GbTub21* was the sole SV-associated *GbTub* gene displaying significant differential expression between accessions harboring versus lacking the corresponding SV. Non-*Tub* neighbors of the candidate hub genes were enriched for cytoskeletal, intracellular-transport, and plasma-membrane functions. **Conclusions**: The pan-genome analysis reveals strong conservation with limited intraspecific variation in the *GbTub* family. Co-expression profiles nominate candidates associated with fiber secondary-wall development, and their causal contribution to fiber strength awaits functional dissection.

## 1. Introduction

Cotton is the world’s most important natural textile raw material, accounting for more than half of the global total of natural fibers [1]. Currently, research on cotton remains primarily focused on increasing yield [2,3]. However, as market demand for cotton fiber evolves, improving quality has become the next major focus for breeders. Fiber strength is a key indicator influencing cotton fiber quality [4]. Cotton fibers are single-cell structures derived from ovule epidermal cells, and their development involves four overlapping stages: fiber initiation (−3 to 3 days post anthesis), rapid elongation (3 to 25 DPA), secondary cell wall synthesis (16 to 40 DPA), and maturation and dehydration (40 to 50 DPA) [1,5,6]. During this process, fiber cells can elongate to 2.5–6 cm, ultimately forming a secondary wall composed almost entirely of cellulose [7,8,9].

Cellulose synthesis, as well as cell division and the stress response, is regulated by microtubules, which therefore serve as a “hub” for plant growth and environmental adaptation [10,11,12]. Microtubules are assembled from tubulin proteins [11,13]. Heterodimers formed by *α-tubulin* (*TubA*) and *β-tubulin* (*TubB*) in a 1:1 ratio assemble into hollow tubular polymers, whereas *γ-tubulin* (*TubG*) participates primarily in microtubule nucleation and organization [14,15,16]. A growing body of research has revealed that *Tub* family genes exhibit developmentally and highly tissue-specific expression patterns in plants [17]. In *Arabidopsis thaliana*, *AtTub1* is primarily expressed in roots and *AtTub5* is enriched in leaves, while *AtTubA1* and *AtTubB9* preferentially accumulate in floral organs [18]. Similarly, in rice, some members are differentially expressed in pollen and vegetative tissues [19]. In woody plants, *Tub* genes are highly expressed in xylem tissues [13]. In eucalyptus, *TubB* xylem tissue expression levels are significantly higher than in leaf tissues [20]. In poplar, *PtTubA1* and *PtTubA5* are expressed in both wood-forming tissues and pollen, whereas *PtTubA6* and *PtTubA8* are specifically expressed only in pollen [21]. This regulation of spatiotemporal expression is essential for microtubule function in specific tissues or developmental stages.

During cotton fiber development, microtubule-associated genes also exhibit distinct stage specificity [15,22]. Co-expression network analyses of cotton fiber development have shown that *Tub* genes are co-expressed with cell wall synthesis-related genes such as expansins, sucrose synthases, cellulases, and pectinases, with expression levels gradually increasing from 5 to 20 DPA [6,23]. Further research revealed that multiple *TubB* genes are preferentially expressed during the fiber elongation stage [24]. A subsequent study revealed that the *GhTub1* promoter is active during both fiber initiation and elongation [17]. Moreover, specific *TubA* genes exhibit unique expression patterns at secondary wall synthesis onset [25,26]. Further research confirmed that there are significant differences in the *TubA* and *TubB* isoform accumulation patterns during fiber development [22]. In addition, *TubB* gene expression is regulated by various plant hormones. Brassinosteroid (BR) treatment regulates cotton fiber development [27]; gibberellin (GA) promotes fiber initiation and elongation [28]; and ethylene induces microtubule reorganization, which also plays a major role in fiber elongation [29]. However, previous studies have been largely confined to *Gossypium hirsutum* [17], whereas the *Tub* gene family in *G. barbadense*, the cultivated cotton species with superior fiber quality, remains poorly characterized in terms of member identification, evolutionary patterns, and functional divergence during secondary wall thickening, limiting the utilization of this elite genetic resource for fiber strength improvement.

In recent years, with the advancement of pan-genomic and cell imaging technologies, significant progress has been made in the functional study of the cotton *Tub* gene family. Further research on the *Tub* genes in *G. barbadense* is therefore of great significance. Traditional gene family studies are constrained by the limitations of a single genome and cannot detect genetic diversity within a species; the concept of the pan-genome was first proposed in bacterial research [30] and has been widely applied in the field of plant science in recent years [31]. Based on a large collection of cotton materials, the first pan-genomes of *G. hirsutum* and *G. barbadense* were constructed, identifying 8851 non-reference genes in *G. barbadense* [32] and elucidating the gene loss patterns following domestication. By integrating 12 newly assembled *G. barbadense* genomes and 17 published allopolyploid genomes, a more refined pan-genome was successfully constructed [1]. Studies on the *G. hirsutum* super pan-genome and the telomere-to-telomere pan-genome have also been conducted [33], capturing a vast number of structural variations and providing a more robust foundation for precision breeding. These studies have collectively provided an unprecedented level of resolution for the cotton pan-genome. Consequently, pan-genomic strategies have been successfully applied in studies of the maize *HSP20* [34] and cassava *HIPP* families [35], revealing genetic variation and functional differentiation that are difficult to capture through traditional single-genome analyses.

To address the limited understanding of intraspecific variation and developmental expression of Tub genes in relation to cotton fiber quality, this study used the *G. barbadense* 3-79 reference genome to identify *GbTub* family members and 12 additional *G. barbadense* accessions to characterize their presence–absence variation, evolutionary constraint, and structural variation (SV). Developmental RNA-seq, RT-qPCR, and transcriptome-wide co-expression analyses were further integrated to identify candidate *GbTub* genes associated with fiber development in the fiber-strength-contrasting accessions 5917 and PimaS-7. By integrating genomic variation, evolutionary conservation, and developmental expression, this study provides a set of prioritized *GbTub* candidates and a model for investigating their potential relationships with fiber secondary-wall development and strength.

## 2. Materials and Methods

### 2.1. Plant Material Preparation and RT-qPCR Analysis

The experiment was conducted at the experimental base of Xinjiang Agricultural Vocational and Technical University, Changji, Xinjiang, China (87.3197° E, 44.0157° N). *G. barbadense* accessions 5917 and PimaS-7, contrasting in fiber strength [36], were grown under the same field conditions. Fiber samples were collected at 0, 5, 10, 15, 20, 25, 30, and 35 DPA, with each replicate pooled from three plants. Samples were immediately frozen in liquid nitrogen and stored at −80 °C.

Total RNA was extracted using the Tiangen Total RNA Extraction Kit for Plants (DP441; Tiangen Biotech, Beijing, China), and cDNA was synthesized using the PrimeScript RT Reagent Kit (RR037A; TaKaRa Bio Inc., Otsu, Japan). Ten representative genes covering distinct expression patterns across 15–35 DPA were selected for RT-qPCR validation. Primers were designed using Primer Premier 5.0, and *GhUBQ7* was used as the internal reference (Table S1). All RT-qPCR reactions were performed with three independent biological replicates and three technical replicates per biological sample to ensure data reliability. Relative expression was calculated using the 2^−ΔΔ*Ct*^ method [37], and RT-qPCR data were analyzed and visualized using GraphPad Prism v8.0.1 (GraphPad Software, San Diego, CA, USA). Two-way ANOVA was used to evaluate the effects of genotype (5917 vs. PimaS-7) and developmental stage on relative gene expression, followed by Tukey’s honestly significant difference (HSD) post hoc test for pairwise comparisons. Statistical significance was defined as *p* < 0.05.

### 2.2. Identification of GbTub Genes in the 3-79 Reference Genome and Phylogenetic Tree Construction

Genome assemblies of 12 *G. barbadense* accessions and the 3-79 reference genome were retrieved from the published pan-genome resource [1] and Figshare (https://figshare.com/projects/Pangenome_of_Gossypium_barbadense/189915; accessed 12 December 2025). *GbTub* candidates were initially identified in the 3-79 reference genome using HMMER (v3.3.2) with the tubulin domain profile PF00091 from Pfam (https://www.ebi.ac.uk/interpro/; accessed 12 February 2026) (E-value ≤ 1 × 10^−5^), validated by BLASTP (v2.12.0) against annotated Tub proteins (E-value < 1 × 10^−10^, identity > 50%, coverage > 70%), and confirmed by NCBI-CDD (https://www.ncbi.nlm.nih.gov/cdd/; accessed 12 February 2026) and Pfam (E-value < 1 × 10^−3^, coverage ≥ 70%). Redundant isoforms were collapsed using CD-HIT (v4.8.1; ≥90% identity), and incomplete sequences and annotated pseudogenes were excluded, yielding 50 non-redundant *GbTub* genes as the reference set. Corresponding accession-level orthologs of these 50 reference-defined genes were subsequently identified in the 12 additional *G. barbadense* genomes.

For phylogenetic analysis, protein sequences of the 50 *GbTub* genes from the 3-79 reference genomes and 17 *A. thaliana* Tub retrieved from TAIR (https://www.arabidopsis.org/; accessed 12 December 2025) were aligned using MAFFT (v7.490). A maximum-likelihood tree was reconstructed using IQ-TREE (v3.1.3) under the Q.INSECT+I+R3 model selected by Model Finder, with 1000 ultrafast bootstrap (UFBoot) replicates (bootstrap values ≥ 70% indicated at nodes). The tree was visualized in iTOL (v6; https://itol.embl.de/; accessed 23 February 2026) as a circular cladogram with uniform branch lengths.

### 2.3. Ka/Ks Calculation

Coding sequences (CDS) and protein sequences of the 50 reference-defined *GbTub* genes and their accession-level orthologs were extracted from the 13 *G. barbadense* genomes. Pairwise Ka and Ks values for accession-level orthologs were calculated using KaKs_Calculator v2.0 (Beijing Institute of Genomics, Chinese Academy of Sciences, China). Ka/Ks distributions were visualized in R (v4.3.1, R Core Team, R Foundation for Statistical Computing, Austria) using ggridges (v0.5.6, Claus O. Wilke, University of Texas at Austin, USA) and ggplot2 (v3.5.0, Hadley Wickham, Posit Software, PBC, USA).

### 2.4. Presence–Absence Variation and SV Association Analysis

Using the 50 reference-defined *GbTub* genes as anchors, presence–absence of their orthologs was assessed across 12 additional *G. barbadense* genomes. Each locus was classified as core (present in all 13 genomes), near-core (12 genomes), accessory (2–11 genomes), or private (1 genome). SV overlapping *GbTub* loci were identified by comparing each accession genome against the 3-79 reference. Deletions, insertions, inversions, and duplications overlapping a *GbTub* gene or its ±2 kb flanking regions were retained and annotated using ANNOVAR (2018-04-16). Pearson correlation analysis was used to evaluate associations between SV presence and gene expression levels. For each SV-associated *GbTub* gene, expression levels were compared between accessions with and without the corresponding SV using a two-tailed unpaired Student’s *t*-test; statistical significance was defined as *p* < 0.05.

### 2.5. Gene Structure, Conserved Motifs, and Prediction of Cis-Regulatory Elements

Conserved motifs in *GbTub* proteins were identified using the Simple MEME Wrapper in TBtools (v1.098, Chen Chengcao, South China Agricultural University, China) with MEME (v5.5.0, Timothy L. Bailey, University of Nevada, Reno, USA). The 2 kb sequences upstream of each transcription start site were submitted to PlantCARE (http://bioinformatics.psb.ugent.be/webtools/plantcare/html/; accessed 25 February 2026) for cis-regulatory element prediction. Gene structures, motifs, domains, and promoter elements were visualized using TBtools.

### 2.6. RNA-Seq Data Analysis

All RNA-seq data were retrieved from our previously published study (GEO accession GSE178945; accessed 25 February 2026) [36]. Raw reads were quality-controlled using FastQC (v0.11.9), trimmed using Trimmomatic (v0.39), and aligned to the *G. barbadense* 3-79 reference genome using HISAT2 (v2.2.1). Gene-level read counts were generated with StringTie (v2.2.1) and used for differential expression analysis in DESeq2 (v1.36.0). Fragments per kilobase of transcript per million mapped reads (FPKM) values were used only for heatmap visualization and expression-trend comparisons. Each sample comprised three independent biological replicates, each with three technical replicates. Differential expression between 5917 and PimaS-7 was assessed at 0, 5, 10, 15, 20, 25, 30, and 35 DPA, with emphasis on 15–35 DPA corresponding to secondary wall thickening. Differentially expressed genes were defined by |log_2_ (fold change) | ≥ 1 and FDR < 0.05.

### 2.7. Co-Expression Network Analysis

The 23 differentially expressed *GbTub* genes were used as seeds for transcriptome-wide co-expression analysis. Pearson correlation coefficients were calculated between each seed and all other expressed genes. For each seed, genes meeting |r| > 0.85 and FDR < 0.01 were retained, and the top 50 partners were selected. These pairs were merged to construct the network, which was visualized using Cytoscape v3.9.1. Among the 23 seeds, the 10 *GbTub* genes with the highest degree centrality were designated candidate hub genes. Their directly connected non-*Tub* neighbors were subjected to GO enrichment analysis using a hypergeometric test against all expressed GO-annotated genes as background (FDR < 0.05).

## 3. Results

### 3.1. Pan-Genomic Identification and Phylogenetic Tree Based on GbTub Genes

**Figure 1:**
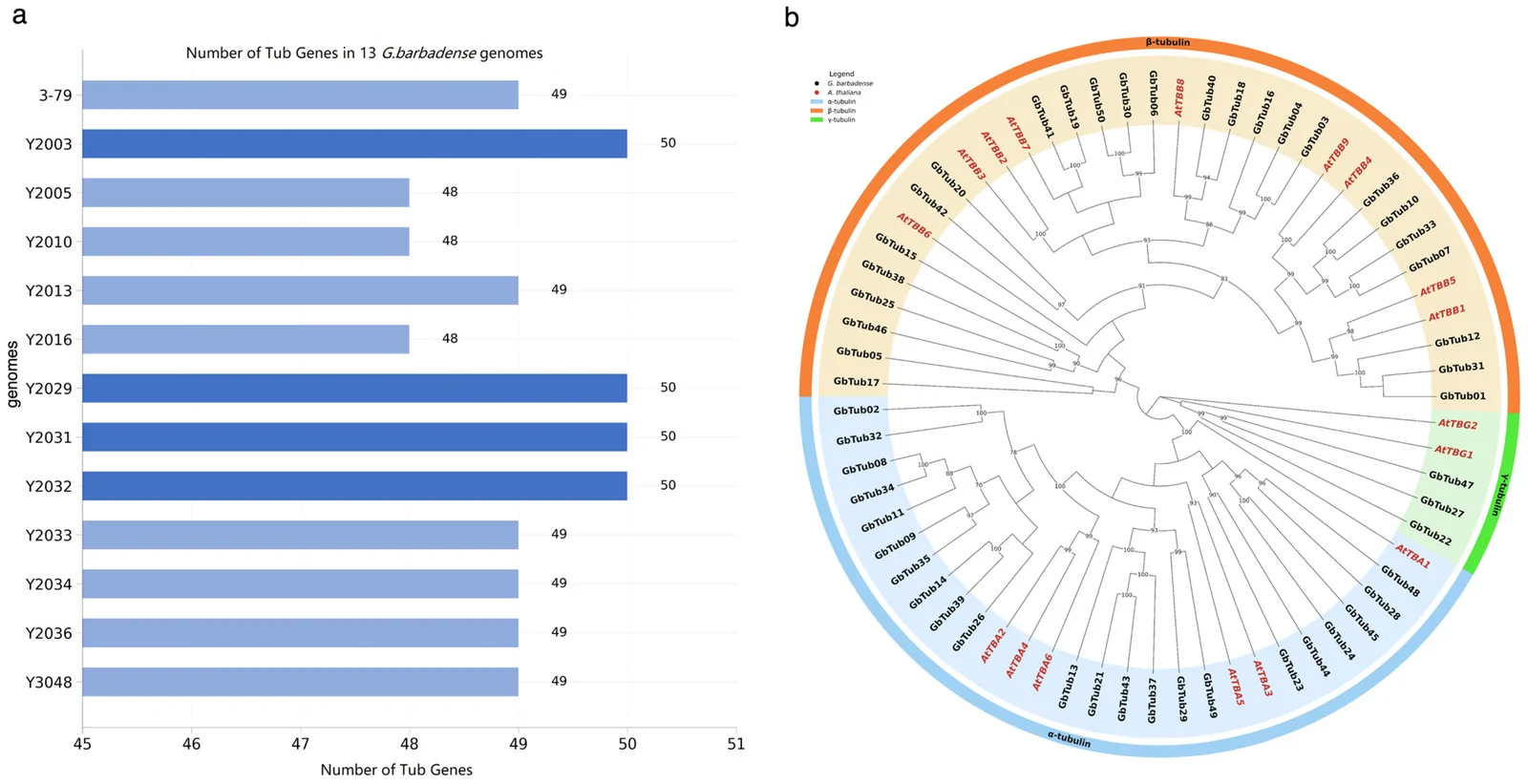
Pan-genome-wide identification and phylogenetic classification of *GbTub* genes. (**a**) Presence–absence variation in the 50 reference-defined *GbTub* genes across the 3-79 reference genome and 12 additional *Gossypium barbadense* genomes. (**b**) Circular cladogram of 50 GbTub proteins from the *G. barbadense* 3-79 reference genome and 17 *Arabidopsis thaliana* Tub proteins. Node labels show ultrafast bootstrap values ≥ 70; branch lengths are not proportional to evolutionary distance. Colors denote *α*- (blue), *β*- (orange), and *γ*-*tubulin* (green) clades.

A total of 50 *GbTub* genes were identified in the *G. barbadense* 3-79 reference genome. Orthologs of these reference-defined genes were subsequently examined across 12 additional *G. barbadense* genomes. Orthologs corresponding to all 50 reference *GbTub* genes were detected in Y2003, Y2029, Y2031, and Y2032, whereas 48 were detected in Y2005, Y2010, and Y2016 (Figure 1a). Based on the orthologous distribution of the 50 reference-defined *GbTub* genes, 43 were classified as core, 6 as near-core, and 1 as accessory; no private gene was detected (Figure S1).

Phylogenetic analysis of the 50 *GbTub* and 17 *A. thaliana* Tub proteins resolved three clades corresponding to *α*- (*TubA*), *β*- (*TubB*), and *γ*- (*TubG*) *tubulins* (Figure 1b). The *β-tubulin* clade was the largest, comprising 34 proteins (25 *GbTub* and 9 *AtTub*), accounting for 50.75% of the total. The *α-tubulin* clade comprised 28 proteins (22 *GbTub* and 6 *AtTub*), accounting for 41.79%. The *γ-tubulin* clade was the smallest, with 5 proteins (3 *GbTub* and 2 *AtTub*), accounting for 7.46%. Both species were represented in all three clades.

### 3.2. Evolutionary Constraint of GbTub Genes

**Figure 2:**
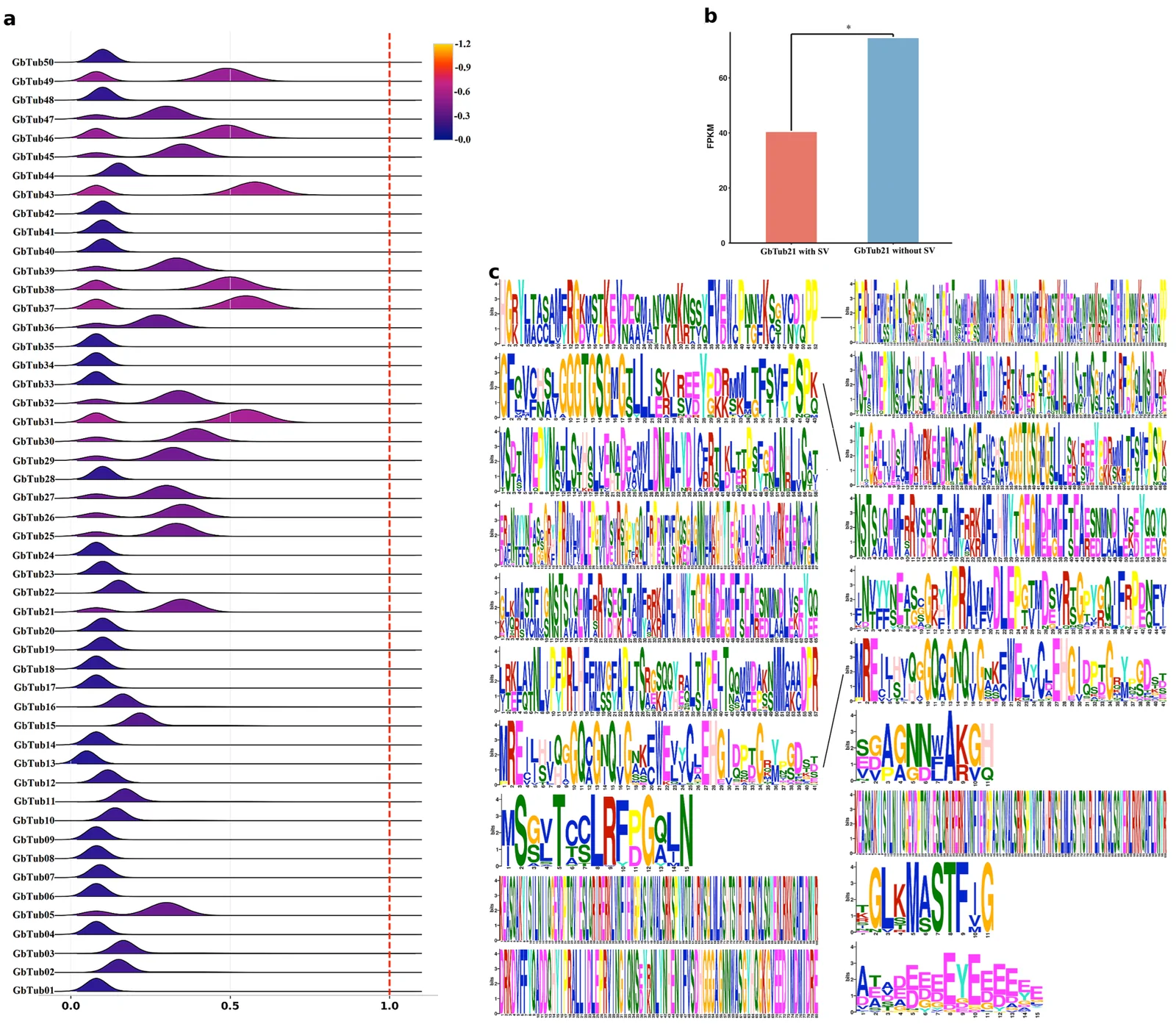
Evolutionary constraint and structural-variation association of *GbTub* genes. (**a**) Ka/Ks distributions across 13 *G. barbadense* genomes. (**b**) *GbTub21* expression in accessions with and without the associated SV (two-tailed unpaired Student’s *t*-test; * *p* < 0.05). (**c**) Sequence alignment of *GbTub21* regions between 3 and 79 and Y2003. Lines indicate corresponding sequence blocks.

Ka/Ks values for accession-level orthologous comparisons across the 13 genomes were below 1 for all 50 reference-defined *GbTub* genes, consistent with predominant purifying selection (Figure 2a). Of these, 30 genes (60%) exhibited a single peak below 0.25, 5 genes (10%) showed a single peak between 0.25 and 0.45, and 15 genes (30%) displayed bimodal distributions with a major peak below 0.25 and a secondary peak at approximately 0.45–0.60. No positive selection signals (Ka/Ks > 1) were detected.

### 3.3. Association of SV with GbTub21 Expression and Gene Structure

Pearson correlation analysis was used to evaluate the association between SV genotypes and gene expression levels. Among the analyzed SV-associated *GbTub* genes, *GbTub21* was the only locus showing a significant expression difference between the two SV-status groups. Accessions carrying the *GbTub21*-associated SV showed significantly lower *GbTub21* expression than accessions lacking the SV (Figure 2b). The *GbTub21*-associated SV was a 3316 bp deletion spanning positions 7,283,740–7,287,055, located 246 bp upstream of the gene. Because Y2003 showed the greatest number of overlapping sequence blocks with the 3-79 reference genome across the *GbTub21* region, it was selected for detailed sequence comparison (Figure 2c). The comparison showed that the *GbTub21* sequences of Y2003 and 3-79 contained three identical conserved domains. Gene structure analysis revealed identical CDS lengths and exon–intron boundaries across all accessions (Figure S2). Conserved-motif analysis further confirmed that Motifs 1–6 were identical across all accessions (Figure S3).

### 3.4. Predicted Cis-Regulatory Elements in GbTub21 Promoters

**Figure 3:**
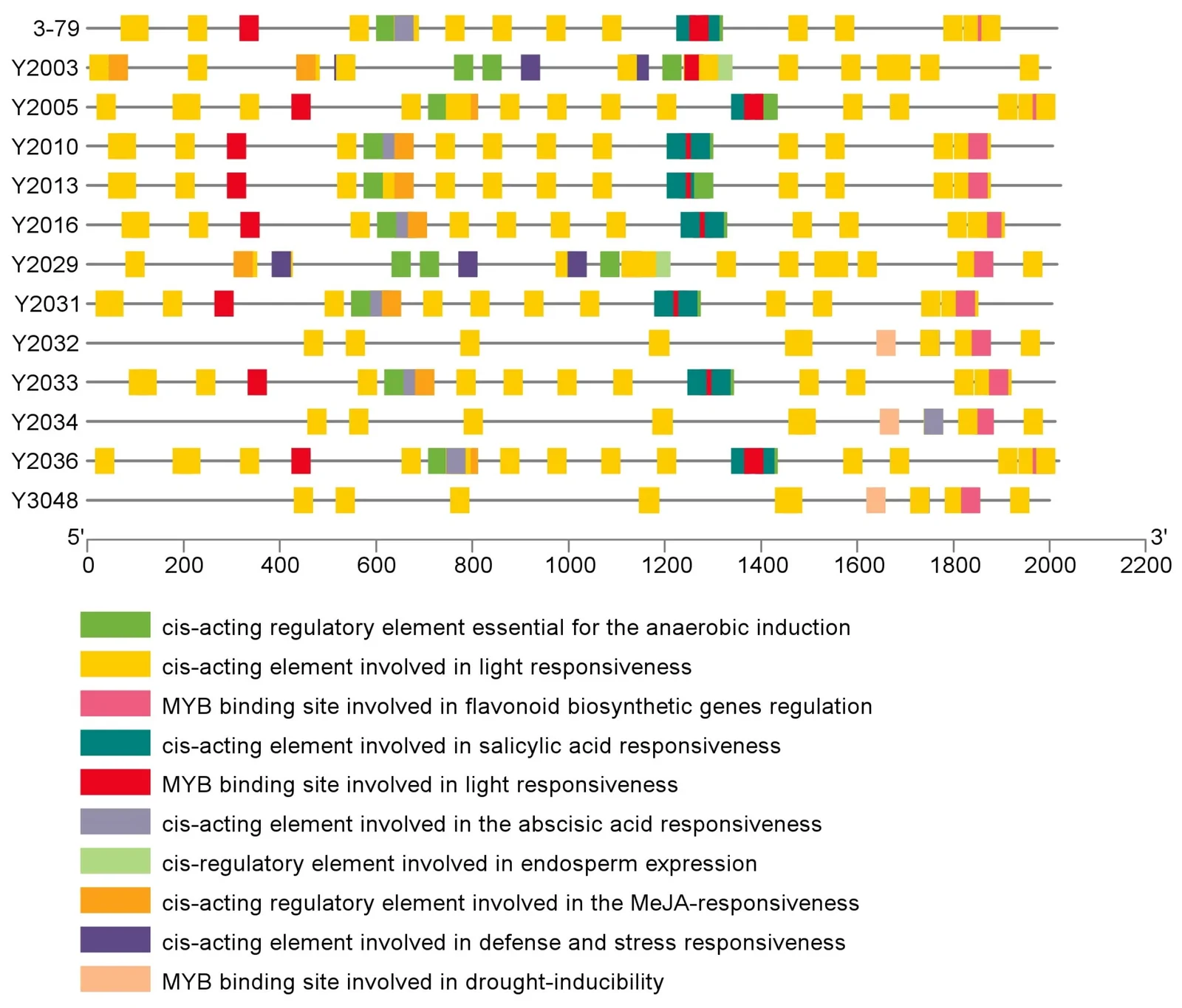
Predicted cis-regulatory elements in the *GbTub21* promoter regions across the examined *G. barbadense* genomes.

PlantCARE analysis identified light-, MYB-, hormone-, and stress-responsive elements within the 2 kb *GbTub21* promoter regions (Figure 3). Light-responsive elements were uniformly enriched across all accessions and spanned the entire promoter region. MYB transcription factor binding sites were widely distributed, including sites associated with light response, flavonoid biosynthesis, and drought induction. Hormone response elements (salicylic acid, methyl jasmonate) were clustered in the 1200–1600 bp region; defense stress, anaerobic induction, and abscisic acid response elements exhibited accession-specific distributions. Endosperm-specific expression elements were present in four accessions (Y2013, Y2034, Y2036, and Y3048). The reference genome 3-79 exhibited the most limited cis-acting element diversity, whereas Y2005, Y2010, and Y2016 possessed a richer array of hormone response elements.

### 3.5. Developmental Expression and Differentially Expressed GbTub Genes

**Figure 4:**
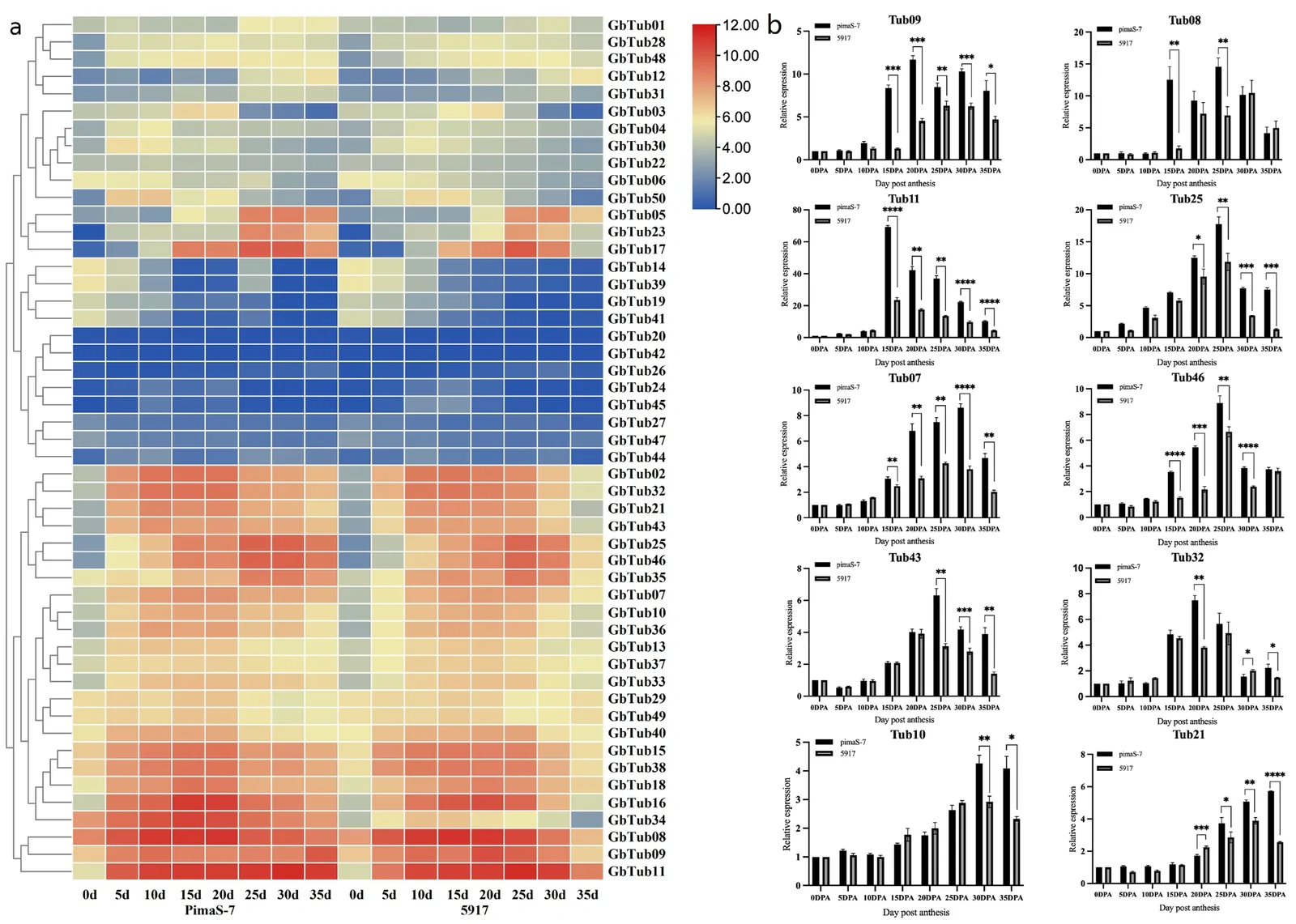
Expression profiles and RT-qPCR validation of *GbTub* genes. (**a**) Heatmap of FPKM-based *GbTub* expression profiles in 5917 and Pima S-7 fibers from 0 to 35 DPA. (**b**) RT-qPCR validation of 10 representative genes selected from the 23 differentially expressed *GbTub* genes. Error bars indicate SD from three biological replicates. Different lowercase letters indicate significant differences between genotypes at the same DPA (two-way ANOVA with Tukey’s HSD post hoc test, *p* < 0.05). * p < 0.05; ** p < 0.01; *** p < 0.001; **** p < 0.0001.

Cluster analysis of FPKM-based expression in 5917 and Pima S-7 fibers at 0–35 DPA divided the *GbTub* genes into two major clusters (Figure 4). One cluster exhibited low expression during fiber elongation (0–10 DPA), whereas the other maintained high expression during secondary wall thickening (15–35 DPA). *GbTub05*, *GbTub23*, *GbTub17*, *GbTub02*, and *GbTub11* were significantly upregulated at 15–35 DPA. Differential expression analysis identified 23 *GbTub* genes that differed significantly between 5917 and Pima S-7 at one or more time points from 15 to 35 DPA (|log_2_(fold change)| ≥ 1 and FDR < 0.05). These genes were retained for subsequent co-expression analysis. Ten representative genes (*GbTub07*, *GbTub08*, *GbTub09*, *GbTub10*, *GbTub11*, *GbTub21*, *GbTub25*, *GbTub32*, *GbTub43*, and *GbTub46*) were validated by RT-qPCR, and their expression trends were consistent with the RNA-seq data (Figure 4b).

### 3.6. Co-Expression Network and Functional Enrichment

**Figure 5:**
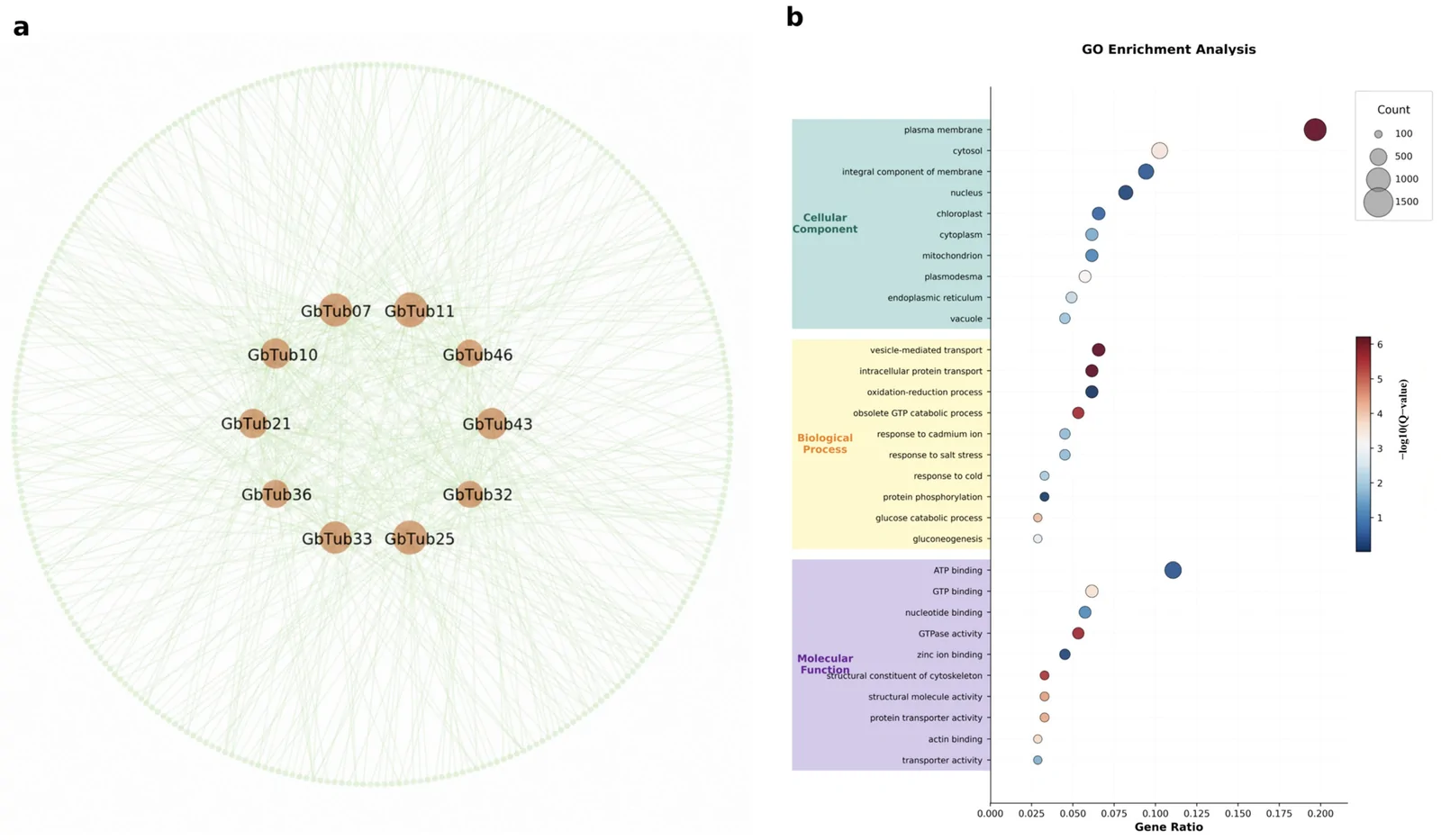
Co-expression network and GO enrichment of candidate hub genes. (**a**) Network of 23 differentially expressed *GbTub* seeds; the 10 candidate hub genes with the highest degree centrality are highlighted. (**b**) GO enrichment of their directly connected non-*Tub* neighbors.

Co-expression analysis identified 10 candidate hub genes based on degree centrality: *GbTub07*, *GbTub10*, *GbTub11*, *GbTub21*, *GbTub25*, *GbTub32*, *GbTub33*, *GbTub36*, *GbTub43*, and *GbTub46* (Figure 5a). GO enrichment of their directly connected non-*Tub* neighbors revealed significant terms (FDR < 0.05; Figure 5b), including cellular component categories (plasma membrane, microtubule, and cell wall), biological process categories (GTP catabolic process, vesicle-mediated transport, intracellular protein transport, and protein polymerization), and molecular function categories (GTP binding, GTPase activity, structural constituent of cytoskeleton, protein transporter activity, and actin binding).

## 4. Discussion

### 4.1. Pan-Genomic Conservation and Evolutionary Constraint

The predominance of core genes (43/50) and the absence of private genes are consistent with strong conservation of the *GbTub* family, although PAV estimates may also be influenced by genome assembly and annotation completeness. The classification of the 50 reference *GbTub* proteins into *α-*, *β-*, and *γ*-*tubulin* clades mirrors the conserved architecture reported in *G. hirsutum* and *A. thaliana* [24]. The modest numerical predominance of *β-tubulin* members in *G. barbadense* is also consistent with the greater number of *β-* than α-*tubulin* isoforms reported in several higher plants [38,39,40,41,42]. Cross-species variation in *α-*, *β-*, and *γ-tubulin* copy numbers may reflect lineage-specific whole-genome and segmental duplication events. Together, the limited intraspecific PAV and phylogenetic conservation support evolutionary constraint within the *GbTub* family.

### 4.2. Structural Conservation and Regulatory Variation

Phylogenetic clustering of *GbTub* genes into *α-, β-*, and *γ-tubulin* subgroups mirrors the conserved architecture reported in *G. hirsutum*, reflecting evolutionary conservation within *Gossypium*. *α-* and *β-tubulins* exhibited highly conserved exon–intron organizations and typical tubulin/tubulin-C domains, consistent with their conserved roles in heterodimer formation and microtubule assembly [16]. By contrast, promoter regions contain diverse predicted cis-acting elements responsive to light, phytohormones, and stresses, suggesting potential transcriptional plasticity. These predictions provide hypotheses for future experimental analysis but do not by themselves demonstrate regulatory activity.

Ka/Ks analysis revealed strong purifying selection across the *GbTub* family, consistent with the conserved functional roles of *Tub*. Notably, *GbTub21* retained conserved CDS length, exon–intron architecture, protein domains, and Motifs 1–6, yet harbored an upstream structural variant associated with expression differences among accessions. The associated SV was a 3316 bp deletion located 246 bp upstream of *GbTub21*; accessions with this deletion exhibited lower *GbTub21* expression than those without it. Given its proximity to the *GbTub21* promoter, this deletion may affect transcriptional regulation. However, this analysis is observational, and genetic-background effects cannot be excluded. Promoter-activity assays or targeted editing will be required to determine whether the deletion directly regulates *GbTub21* transcription.

### 4.3. Developmental Expression and Functional Framework

The differential expression of 23 *GbTub* genes between 5917 and PimaS-7 at 15–35 DPA spans the transition from late fiber elongation to secondary wall thickening. This temporal association between cytoskeletal dynamics and cell-wall deposition is consistent with the microtubule guidance hypothesis [9,26]. During fiber development, cortical microtubules are associated with the organization of cellulose synthesis and microfibril orientation [8,20], while microtubule-associated proteins can interact with *Tub* to influence fiber elongation [43]. Comparative transcriptomic studies in *G. hirsutum* have similarly identified *Tub* genes that are differentially expressed during fiber development [24], suggesting that *Tub* expression dynamics may be relevant to fiber developmental transitions across *Gossypium* species. Nevertheless, the expression differences observed here remain correlative and do not establish a causal contribution to fiber-strength variation.

Co-expression analysis identified 10 candidate hub genes whose directly connected non-*Tub* neighbors were significantly enriched in cytoskeletal organization, intracellular transport, plasma-membrane functions, vesicle-mediated transport, actin binding, and protein polymerization. These enrichments are consistent with established links among cytoskeletal organization, intracellular trafficking, and cellulose-synthase delivery [44]. Within this context, the candidate hub genes may be associated with microtubule-dependent intracellular transport and plasma-membrane processes relevant to cellulose deposition. The correlational associations identified in this study align with the well-documented function of cortical microtubules in cellulose deposition and provide guidance for subsequent functional validation. The 10 candidate hub genes show differential expression during 15–35 DPA, when cellulose deposition largely determines fiber strength [9,26]; while this remains correlative, the contrasting expression between 5917 and PimaS-7 points to potential breeding targets for improving fiber quality and textile performance.

### 4.4. Limitations and Future Directions

The expression and co-expression analyses were based on a single transcriptome dataset from our previous study and two accessions, limiting generalizability. Possible functional redundancy among At and Dt-subgenome homologs remains unresolved. The comparison of two accessions provides only a correlation; it cannot establish whether observed expression differences causally contribute to fiber-strength variation or reflect broader genetic-background effects. Pan-genome analysis depends on the completeness of genome assembly and the accuracy of gene annotation, which may introduce potential biases. The functional roles inferred here remain to be validated through direct genetic perturbation. Future studies may adopt CRISPR/Cas9 gene editing, virus-induced gene silencing (VIGS), and yeast two-hybrid assays to dissect the molecular functions of key *GbTub* genes and their regulatory networks. Integrating these functional characteristics with genomic selection and marker-assisted breeding for fiber strength traits may provide promising targets for the genetic improvement of cotton fiber quality.

## 5. Conclusions

A total of 50 *GbTub* genes were identified in the *G. barbadense* 3-79 reference genome, and their ortholog distribution across 12 additional accessions classified 43, 6, and 1 as core, near-core, and accessory genes, respectively. Ka/Ks analysis revealed pervasive purifying selection across the family. Notably, *GbTub21* harbors an accession-specific structural variant linked to differential expression. Co-expression profiles nominate candidate hub genes for secondary-wall development, aligning with known microtubule functions in cellulose deposition, and their causal contribution to fiber strength awaits functional dissection.

## Data Availability

All datasets generated during the current study are included in the main text or available in the online Supplementary Materials.

## Supplementary Materials

The following supporting information can be downloaded at: https://www.mdpi.com/article/10.3390/genes17080873/s1, Figure S1. Heatmap showing the presence and absence of *GbTubs* in 13 *G. barbadense* genomes. Figure S2. Comparison of exon-intron structures of *GbTub* genes among various *G. barbadense* accessions. Figure S3. Comparative analysis of conserved motifs 1–6 of *GbTub* proteins among *G. barbadense* accessions. Table S1: All primer sequences of qRT-PCR.
