# Supplementary material for: Pan-Genome Analysis of the Tubulin Gene Family Reveals Candidates for Fiber Strength in Gossypium barbadense
Source: Genes (Basel). 2026 Jul 27;17(8):873. doi: 10.3390/genes17080873 (PMC13512239; doi:10.3390/genes17080873)
Supplement: Supplementary file 1 [file genes-17-00873-s001.zip › Supplementary Figures S1-S3 .pdf]

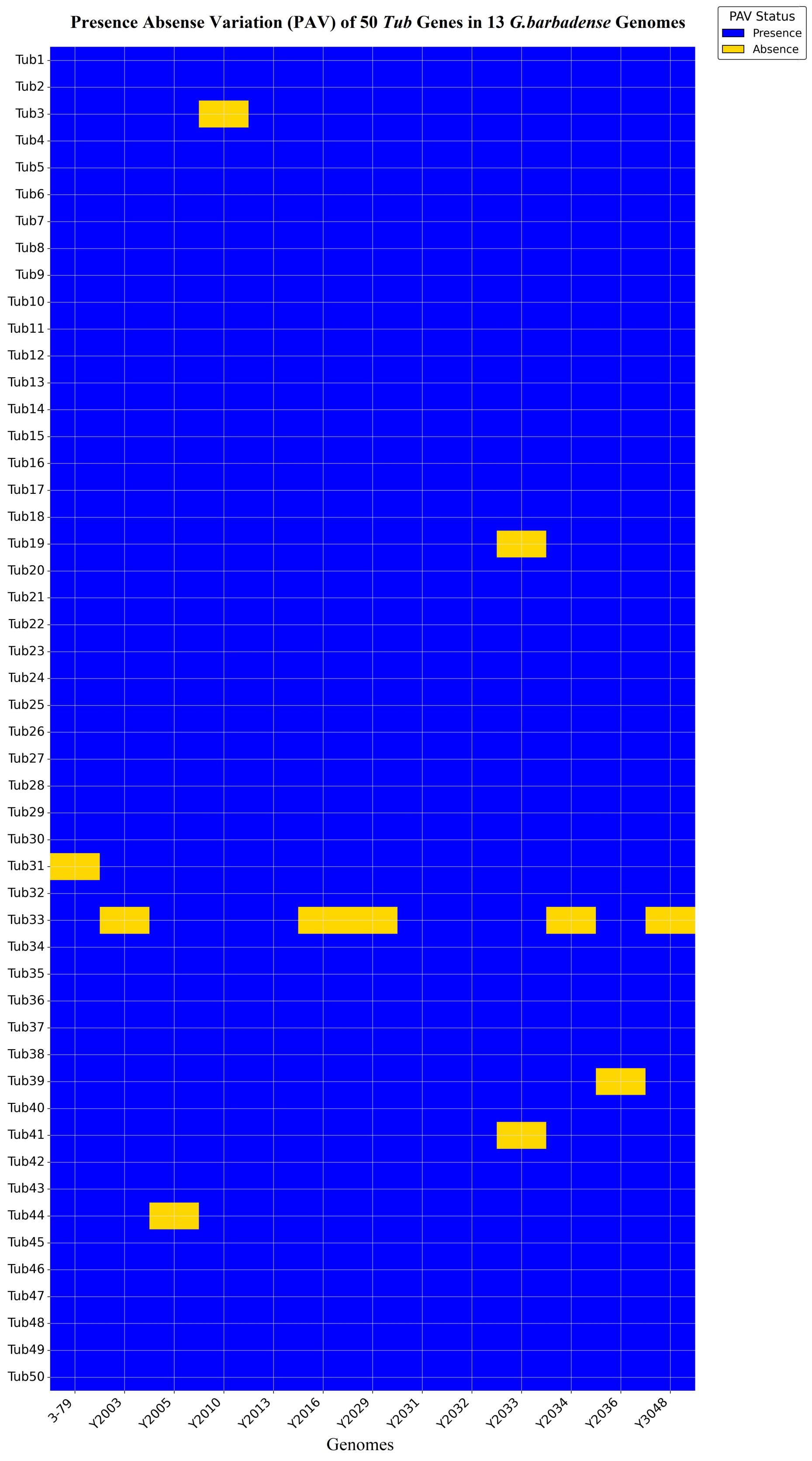

Figure S1. Heatmap showing the presence and absence of *GbTubs* in 13 *G. barbadense* genomes.

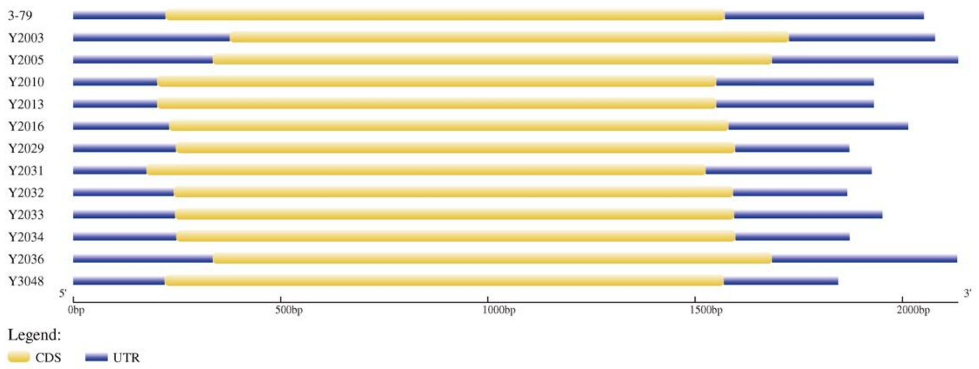

Figure S2. Comparison of exon-intron structures of *GbTub* genes among various *G. barbadense* accessions.

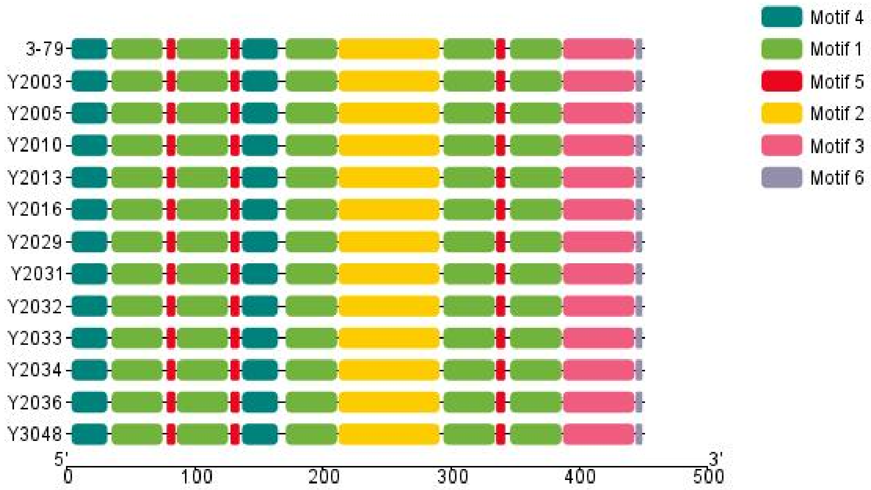

Figure S3. Comparative analysis of conserved motifs 1— 6 of *GbTub* proteins among *G. barbadense* accessions.
